# The first human trial of CRISPR-based cell therapy clears safety concerns as new treatment for late-stage lung cancer

**DOI:** 10.1038/s41392-020-00283-8

**Published:** 2020-08-25

**Authors:** Shenghui He

**Affiliations:** 1 The Lineberger Comprehensive Cancer Center, University of North Carolina School of Medicine, Chapel Hill, NC 27599-7295 USA; 2 Department of Genetics, University of North Carolina School of Medicine, Chapel Hill, NC 27599-7295 USA

**Keywords:** Immunotherapy, Lung cancer, Genetic engineering

In a study recently published in *Nature Medicine*, Lu et al. examined the feasibility and safety of using CRISPR (Clustered Regularly Interspaced Short Palindromic Repeats)-engineered, patient-derived T cells to treat late-stage lung cancer.^1^

Gene-editing therapy holds great promise in treating a wide range of human diseases from cancer to genetic disorders. The introduction of the CRISPR technology, due to its simplicity and intrinsic programmability,^2^ has revolutionized the gene-editing field, and quickly surpasses both zinc-finger nuclease and transcription activator-like effector nuclease to become the method-of-choice for therapeutic gene editing.^3^ However, due to potential off-target effects, the safety of CRISPR-based therapy remains controversial. In a study recently published in *Nature Medicine*,^1^ Lu et al. examined the feasibility and safety of using CRISPR-engineered T cells to treat late-stage lung cancer. They demonstrated very low off-target editing rates and no severe treatment-related adverse events (AE), thus supporting its general safety for clinical use.^1^

Non-small cell lung cancer (NSCLC) accounts for 83% of all lung cancer and is the leading cause of cancer-related death worldwide. Currently, immune checkpoint inhibitors targeting either PD-1 or PD-L1 have become part of the standard treatment for late-stage, PD-L1-expressing NSCLC with no molecular drivers. To determine whether PD-1-edited autologous T cells can be a viable alternative to antibody-based immunotherapy such as pembrolizumab in cancer treatment and to examine the general safety and feasibility of CRISPR-based cell therapy, Lu and colleagues conducted the first-in-human trial of CRISPR-edited, *PD-1*-ablated T cells in patients with advanced NSCLC. In total, 12 patients received transfusion of edited T cells and were monitored for up to 96 weeks for treatment-related AEs.

**Fig. 1:**
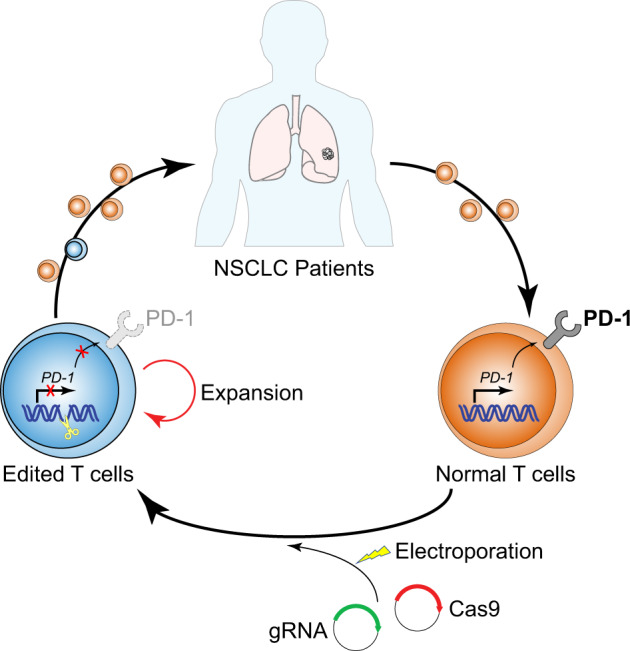
Study design by Lu et al. PBMCs were isolated from late-stage NSCLC patients and electroporated with plasmids encoding Cas9 and a pair of gRNAs targeting the second exon of *PD-1* gene. The edited T cells were expanded in vitro for 17–40 days before being reinfused back into patients. Treated patients were monitored for up to 96 weeks for in vivo persistence of edited T cells, treatment-related AEs, and disease progression

To generate PD-1-ablated T cells, the authors electroporated plasmids encoding Cas9 and guide RNAs (gRNA) targeting the second exon of the *PD-1* gene into patient-derived peripheral blood mononuclear cells (PBMCs). The transfected cells were expanded ex vivo for 17–40 days before being reinfused into patients in a dose-escalation study (Fig. 1). The editing efficiency and in vivo persistence of edited T cells were monitored by next-generation sequencing (NGS) of mutations at the *PD-1* locus in infused cells for up to 1 year. The authors observed relatively low levels of editing efficiency (median = 5.81%) in infused cells, which may be related to the use of older-generation plasmid-based gene delivery system instead of the newer more efficient ribonucleoprotein method at the time of the study. As a result, edited T cells did not persist long term in most patients.

To evaluate the impact of off-target editing by CRISPR-Cas9 in edited T cells, the authors performed both targeted NGS and unbiased whole-genome sequencing (WGS) to identify the type and frequency of off-target mutations. Using high coverage (20,000×) NGS, only an average of 0.05% mutation rate was detected in 18 potential off-target sites. In contrast, no mutations were detected in 2086 predicted off-target sites using the lower coverage (100×) WGS method. Taken together, these results suggested off-target editing by CRISPR was rare. Infused patients were monitored for up to 2 years for treatment-related AEs. Grade 1/2 AEs occurred in 11 of the 12 patients and no grade 3+ AEs were reported. No treatment-related dose-limiting toxicities were observed during the 2-year follow-up period, suggesting infusions of *PD-1*-edited T cells were well-tolerated. The authors did not observe objective response in the treated patients, which may be due to a combination of low editing efficiency, poor T cell expansion, and/or lack of antigen specificity by the edited T cells. Of note, one patient with the highest *PD-1* editing efficiency showed long-term persistence of edited T cells in vivo and stable disease for 76 weeks, suggesting higher editing efficiency may be associated with improved clinical outcomes.

Together, this study showed that using ex vivo CRISPR-edited, *PD-1*-ablated, patient-derived T cells for cancer treatment is clinically feasible and generally safe. This finding echoes the conclusions of two other recent clinical studies that also examined the safety and feasibility of CRISPR-based cell therapy. In the first study, Xu et al. transplanted CRISPR-engineered, *CCR5*-ablated hematopoietic stem/progenitor cells into a patient with HIV infection and acute lymphoblastic leukemia. The authors found the edited donor cells to persist more than 19 months after transplantation without causing gene-editing-related AEs.^4^ In the second study, Stadtmauer et al. used CRISPR-based multiplex gene editing to ablate the expression of endogenous T cell receptor (*TCR*) and *PD-1* genes in T cells engineered to express a cancer-specific *TCR* gene in four patients. The authors reported efficient editing of all target genomic loci and stable engraftment of edited T cells in vivo for up to 9 months. While chromosome translocations were detected in the edited cells as would be expected from multiplex editing, no cellular transformation was observed and patients experienced no gene-editing-related severe AEs.^5^ In both studies, the rates of off-target editing were low.

These three studies marked an important milestone in the development of CRISPR-based gene therapy. Not only that they provided the eagerly awaited first pieces of evidence supporting the safety and feasibility of CRISPR-based cell therapy in human patients, but also that they established detailed protocols for CRISPR-editing and off-target events tracking in clinical settings which are valuable for designing future trials. Nevertheless, these studies also revealed several limitations of existing gene-editing technology. First, the gene-editing efficiency varies significantly across target genes and patients.^1,5^ This may be due to the different cell production protocols used in separate studies, but it emphasizes the importance of optimizing and validating each individual CRISPR assay for clinical use. Second, while the reported off-target rates were low in all three studies, they were highly dependent on gRNA selection, and multiplex gene editing significantly increased the risk of chromosome translocation.^5^ Given that adverse effects of off-target editing may take several years to manifest,^3^ it is important for future studies to evaluate the long-term safety of CRISPR, particularly when the edited cells are long-lived in vivo. Finally, the current protocols for producing patient-derived CRISPR-edited cells are still somewhat inefficient. Notably, in two studies, the authors failed to expand enough edited T cells from a significant fraction of patients (23%^1^ and 33%,^5^ respectively). While this may be because these patients were heavily treated prior to T cell isolation, it does reveal potential limitation of current approach in clinical applications.

Some of these limitations may be addressed by newer generation of CRISPR-based gene-editing technologies such as “base editing” and “prime editing”.^2^ Unlike the standard CRISPR-Cas9 approach used in the current study that relies on generating random indels to disrupt target gene function, these newer methods are designed to generate uniform and predictable gene-editing results, thus improving editing precision.^2^ More importantly, they do not create double-strand DNA breaks, thus having the potential to significantly improve the safety of therapeutic gene-editing by avoiding generating one of the most toxic form of DNA damage in mammalian cells.^2^ Currently, these methods remain at early stage of pre-clinical development as their efficiency and specificity are still being assessed. Nevertheless, the valuable insights gained from the current batches of CRISPR-based trials by Lu et al. and others will facilitate the design of future trials based on these new technical advances.
